# Genomic Deregulation of the E2F/Rb Pathway Leads to Activation of the Oncogene EZH2 in Small Cell Lung Cancer

**DOI:** 10.1371/journal.pone.0071670

**Published:** 2013-08-15

**Authors:** Bradley P. Coe, Kelsie L. Thu, Sarit Aviel-Ronen, Emily A. Vucic, Adi F. Gazdar, Stephen Lam, Ming-Sound Tsao, Wan L. Lam

**Affiliations:** 1 Integrative Oncology Department, BC Cancer Research Centre, Vancouver, Canada; 2 Pathology Department, Sheba Medical Centre, Tel-Hashomer, Israel; 3 Hamon Center for Therapeutic Oncology Research and Department of Pathology, University of Texas Southwestern Medical Center, Dallas, Texas, United States of America; 4 Department of Laboratory Medicine and Pathobiology, University of Toronto, Toronto, Canada; 5 Department of Pathology, Princess Margaret Hospital University Health Network, Toronto, Canada; H. Lee Moffitt Cancer Center & Research Institute, United States of America

## Abstract

Small cell lung cancer (SCLC) is a highly aggressive lung neoplasm with extremely poor clinical outcomes and no approved targeted treatments. To elucidate the mechanisms responsible for driving the SCLC phenotype in hopes of revealing novel therapeutic targets, we studied copy number and methylation profiles of SCLC. We found disruption of the E2F/Rb pathway was a prominent feature deregulated in 96% of the SCLC samples investigated and was strongly associated with increased expression of EZH2, an oncogene and core member of the polycomb repressive complex 2 (PRC2). Through its catalytic role in the PRC2 complex, EZH2 normally functions to epigenetically silence genes during development, however, it aberrantly silences genes in human cancers. We provide evidence to support that EZH2 is functionally active in SCLC tumours, exerts pro-tumourigenic functions *in vitro*, and is associated with aberrant methylation profiles of PRC2 target genes indicative of a “stem-cell like” hypermethylator profile in SCLC tumours. Furthermore, lentiviral-mediated knockdown of EZH2 demonstrated a significant reduction in the growth of SCLC cell lines, suggesting EZH2 has a key role in driving SCLC biology. In conclusion, our data confirm the role of EZH2 as a critical oncogene in SCLC, and lend support to the prioritization of EZH2 as a potential therapeutic target in clinical disease.

## Introduction

Small cell lung cancer (SCLC) is a highly aggressive lung neoplasm with an extremely poor clinical outcome, which has seen little improvement over the past 25 years [1]. Due to its unique clinical course, SCLC is often staged using a separate clinical staging system from the standard TNM system used for most cancers including all non-small cell lung cancers (NSCLC), as either limited (33%) or extensive (67%) stage disease based on the extent of tumour spread. Patients with limited stage disease have a median survival of 18 months, while that for patients with extensive stage is only 9 months [2]–[4]. Due to its highly aggressive nature, surgery is rarely performed and chemotherapy with or without concurrent radiotherapy is the usual treatment. Despite SCLC initially presenting as a chemo-sensitive disease, almost all patients relapse after initial treatment, and no targeted therapeutics have been approved for SCLC to date [5]–[9]. Therefore new targets for therapeutic intervention are urgently needed for this disease.

The identification of oncogenes which are selectively activated and that functionally drive tumour phenotypes, make ideal therapeutic targets for patients harbouring these aberrations. In NSCLC, the discovery of such events has led to the development and application of targeted chemotherapies, translating to improved progression free and overall survival for NSCLC for selected patients [10]. Recent genomic profiling towards this aim in SCLC have uncovered thousands of mutations and deletions, occurring in multiple tumour suppressor pathways (eg. *TP53*, *RB1*, *PTEN*, *EPHA7*), and genes involved in chromatin modification (eg. *CREBBP*, *MLL*), as well as amplifications in putative SCLC cancer driver genes, such as *SOX2* [5], [8]. These findings are significant and may lead to the development and application of novel targeted therapies for very specific subgroups of SCLC patients, however the elucidation of a more ubiquitously activated target in SCLC would be even more beneficial.

DNA level inactivation of the TSG *RB1* is nearly universal in SCLC. RB1 normally inhibits proliferation through inhibition of the E2F transcription factors. E2F family members are commonly overexpressed in various tumours, including SCLC [11]–[15]. Work by us and others has identified DNA level gains of *E2F* family members in SCLC cells lines, tumours and murine models [5], [16]. EZH2, a core member of the polycomb repressive complex 2 (PRC2), is a downstream target of the Rb1/E2F complex. EZH2 is critical to the epigenetic maintenance of embryonic cell “stem-ness” and the establishment of lineage specificity during normal development. *EZH2* has recently been established as a driver oncogene, where it is involved in the aberrant hypermethylation of tumour suppressor genes (TSG) in multiple cancer types. Overexpression of EZH2 was recently described in SCLC, where it has been proposed as a novel SCLC therapeutic target [11].

Mechanisms underlying EZH2 hyperactivity have been identified in other cancers and include mutation of *EZH2* itself which causes over-activation of its histone modifying capacities and DNA hypermethylator phenotypes that are associated with poor outcome, chemo- resistance and highly aggressive tumour phenotypes. To explore mechanisms driving EZH2 activation and to confirm the oncogenic role of EZH2 in SCLC, we studied copy number profiles of 14 SCLC tumours and 14 SCLC cell lines and assessed cell viability after EZH2 knockdown. We found that DNA level disruption of E2F/Rb pathway components, by either loss of *RB1* or amplification of *E2F*, occurred in 100% of the SCLC samples investigated, which we found strongly associated with increased expression of EZH2. We confirm a functional oncogenic role for EZH2 in SCLC, and an associated and DNA hypermethylator profile for SCLC tumours. We propose that the genomic disruption of E2F/Rb leads to activation of the histone methyltransferase EZH2 which functions as an oncogene in SCLC, further supporting its promise as a therapeutic target for SCLC patients.

## Methods

### Sample Procurement and Array Comparative Genomic Hybridization

A great majority of SCLC patients are not surgical candidates, thus only archival biopsy samples are available. A panel of 14 formalin-fixed and paraffin embedded SCLC tumour samples were obtained from the archive of the University Health Network (UHN) Department of Pathology, with a study protocol approved by the UHN Ethics Board, which did not require specific informed consents for materials from prior to 2000 and patients who were deceased (Table S1). Tissue cores were selected from regions of tumour blocks which demonstrated sufficient purity for a 2 mm core. After histological review, samples were extracted by standard xylene based deparaffinization and standard proteinase K based DNA extraction protocol with the addition of base hydrolysis (10 minute 70 C incubation with ½ volume 1M NaOH followed by neutralization with ½ volume 1M HCl) to remove any contaminating RNA. Array CGH was performed on a platform amenable to FFPE material, and copy number calls generated as previously described [16], [17].

### Quantification of E2F and EZH2 Expression Levels

RT-PCR was performed using TaqMan gene expression assays with standard protocols on an ABI 7500 Fast thermocycler (Applied Biosystems, Foster City, CA, USA). The TaqMan assays used were E2F1 (Hs00153451_m1), E2F2 (Hs00231667_m1), E2F3 (Hs00605457_m1), Rb (Hs00153108_m1), EZH2 (Hs00172783_m1), and 18S RNA (HS99999901_s1). Absolute expression values were calculated for E2F1, E2F2, E2F3 and RB1 by scaling the delta Ct values (gene-18s) to a value between 0 and 1000. Protein levels of EZH2 and E2Fs in cell lines were assessed using standard Western blotting methods (Cell Signaling primary antibodies: #3147 (EZH2) and #2118 (GAPDH); Santa Cruz primary antibodies: sc-251 (E2F1), sc-632 (E2F2), and sc-878 (E2F3)) [18]. Band intensities were quantified using ImageJ software. Immunohistochemistry (IHC) was performed using standard techniques with a primary anti-EZH2 antibody (rabbit polyclonal, 18-7395, Life Technologies, Grand Island, NY). Images were scored based on the intensity of staining observed on a scale from 0 (low) to 3 (high).

### DNA Methylation Analysis

We obtained DNA methylation profiles for 12 SCLC tumours, and 21 control bronchial small airway epithelial (SAE) specimens using Illumina’s Infinium Human Methylation (HM27) assay. SAE were obtained by bronchial brush during routine bronchoscopy from small airways (defined as <2 mm in diameter) of individuals without presence or prior history of lung cancer or chronic obstructive pulmonary disease [19]. DNA samples were bisulfite converted using the Zymo EZ DNA Methylation bisulfite conversion kit (Zymo Research Corporation, Orange, CA) and HM27 profiles generated as previously described [20]. Infinium HM27 methylation sentrix array files (.sdf) and.idat image files were loaded into Illumina GenomeStudio. Raw data were exported and read into R: A Language and Environment for Statistical Computing, and corrected for red green color channel bias and batch effect between multiple methylation experiments, using the Bioconductor package lumi, which applies a color correction and SSN normalization algorithm [21]. Probes with an Illumina array detection p<0.05, present in >58% of cases in each group were retained. Percent methylation for each probe is presented as a β values (max(yi,methy,0)/(max(yi,unmethy,0)+max(yi,methy,0)) +100). A list of EZH2 bound targets in embryonic mouse stem cells was obtained from a publication by Ku *et *al [22]. Targets were aligned to orthologous human genes using the Mouse Genome Database [23]. To identify EZH2 targeted genes that were differentially methylated between SCLC and normal groups, a non parametric permutation tests using 10,000 permutations was performed as described by Chari *et al*. [24]. An M-value was calculated by the log_2_ ratio of methylated probe intensity over unmethylated probe intensity, and used as input for the permutation test [25]. Methylation permutation scores were corrected for multiple testing using the Benjamini and Hochberg (B-H) method. Average β values for SCLC tumours were subtracted from average β values from normal airway controls to obtain a delta β value. Hypermethylated and hypomethylated probes in SCLC tumours were defined as those with delta β values > = 0.2 or< = - 0.2, respectively. Probes that fulfilled the following criteria: *i*) permutation B-H corrected p<0.05, *ii*) a delta beta value> = 0.2 or< = –0.2 and *iii*) a standard deviation (SD) ≤2 within each group, were considered differentially methylated (DM) between SCLC tumours and normal airways.

### Correlation between EZH2 and E2F Expression Levels

To determine the association between *EZH2* and *E2F1*, *E2F2*, *E2F3*, and *RB1* mRNA expression levels in SCLC samples, we queried publically available expression data from Peifer et al. [5] and the Broad Institute's Sanger Cell Line project (broadinstitute.org/cgi-bin/cancer/datasets.cgi). GraphPad Prism 6 was used to calculate Spearman correlation coefficients between *EZH2* and the E2F/Rb pathway genes.

### Knockdown of *EZH2 and E2F* in SCLC lines and *E2F* Expression in HBEC Cells

293T, HTB-175 and H524 SCLC cell lines were obtained from the ATCC and grown according to ATCC instructions. Immortalized human bronchial epithelial cells (HBEC) were grown in KSFM media supplemented with bovine pituitary extract and EGF. Lentiviral PLKO-puromycin resistant plasmids encoding shRNAs targeting *E2F1*, *E2F2*, *E2F3*, and *EZH2* were purchased from Open Biosystems (RHS3979-201768515, RHS3979-201745380, RHS3979-201745384, and RHS4533-NM_004456). Lentiviruses were generated as previously described [26]. Multiplicity of infection (MOI) was determined for both the PLKO (empty vector control) and E1 (EZH2 targeting shRNA) viruses using custom TaqMan primers (forward 5' - GCGGTGTTCGCCGAGAT - 3' and reverse 5' - GAGGCCTTCCATCTGTTGCT - 3'). Transfection was performed as described in Lockwood et al [26]. Briefly, for each line and virus 150,000 cells were infected (using an MOI of 15 for EZH2 viruses) in media supplemented with 2 ug/ml polybrene. Growth media was supplemented with 1 ug/ml puromycin to select for infected cells. To assess the viability of *EZH2* knockdown relative to controls cells MTT assays were performed as previously described [26]. *E2F1* (EX-Z8212-M46), *E2F2* (EX-F0378-M46), *E2F3* (EX-T0731-M46), and luciferase control (EX-hLUC-M46) ORF expression clones were purchased from GeneCopoeia. Transient expression experiments were performed using Lipofectamine LTX following the manufacturer's instructions. Efficiency of E2F knockdown and overexpression, and their effects on EZH2 expression levels were determined by Western blotting.

## Results and Discussion

### Genomic Profiling of SCLC Tumours, and Comparison with SCLC Cell Lines

Details of the DNA copy number profiles of these tumours along with 14 unrelated SCLC cell lines are presented in Figure S1, and data are available in the Gene Expression Omnibus. Strikingly, we observed recurrence of DNA copy number alterations in SCLC tumours, including regions encompassing top pathway candidates from our previous cell line study such as *TCF4* (18q21), *STMN1* (18q21) and *E2F2* (1p36) [16].

### The E2F/Rb Pathway is Specifically Deregulated in SCLC

Based on previous observations by us and others, we hypothesized that cell cycle activation in SCLC may occur as far downstream as the transcription factors that regulate cell cycle progression. The retinoblastoma pathway has long been established as a key target of deregulation in SCLC. *RB1* is frequently lost or mutated (60–90%) in SCLC and demonstrates reduced expression in the majority of cases [5], [8], [11]–[13], [27]. A primary function of Rb is its normal inhibition of the E2F transcription factors. When Rb1 is inactivated by phosphorylation, E2F proteins are free to function as transcription factors activating a collection of cell cycle progression factors. The E2F family of genes is divided into activating (E2F1, E2F2, E2F3) and inhibitory (E2F4, E2F5) members which physically interact with Rb1 [15], [27]–[31].

Recent evidence has demonstrated the E2F genes may also be primary targets of deregulation. Other studies have detected high expression levels of E2F1 and E2F3 in various tumours, including SCLC [11]–[15]. Additionally, Peifer et al noted high level DNA amplification of *E2F2* in mouse models of SCLC [5]. Taken together with our own observations of *E2F2* copy number gain and over expression specifically in SCLC cell lines and validation of *E2F2* copy number alterations in SCLC tumours, we decided to further analyze this pathway in the context of copy number and transcriptional deregulation.

**Figure 1:**
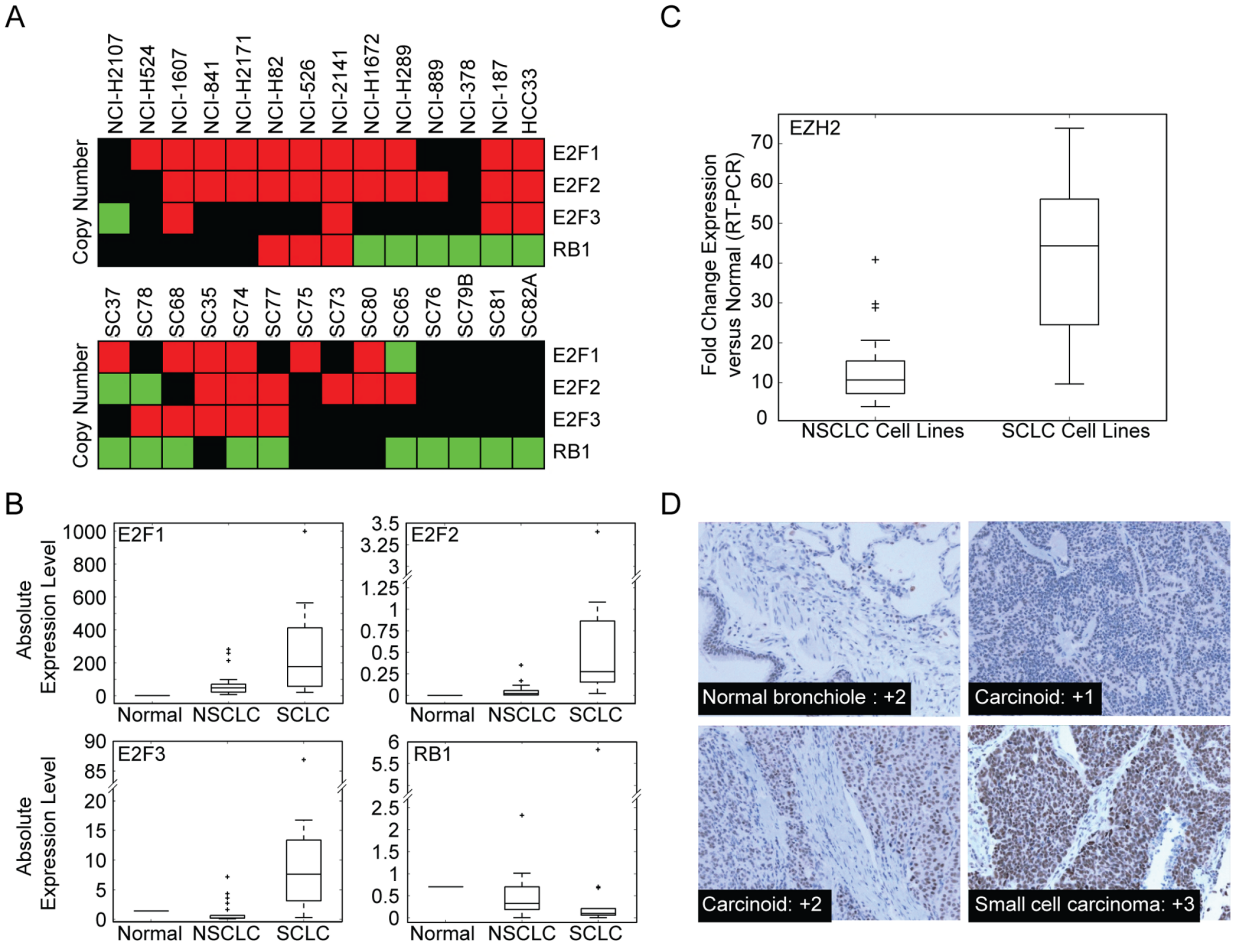
Deregulation of the E2F transcription factors. (**A**) Copy number alterations of specific E2F/Rb pathway members in SCLC cell lines (top) and tumours (bottom). Green shading represents loss, while red represents gain and black represents no change. (**B**) Expression of E2F/Rb pathway members by RT-PCR. Data are presented as box-plots of absolute expression levels derived from scale normalized PCR data. The centre line in each box represents the median level while the box represents the interquartile range, with whiskers extending to the last non-outlier data point (defined as 1.5× the interquartile range). Outliers are represented as crosses. (**C**) Significant overexpression of EZH2 was observed in SCLC compared to NSCLC samples, coinciding with excess activation of the E2F/Rb pathway. (**D**) IHC was performed using standard techniques with a primary anti-EZH2 antibody and images were taken at 200× magnification. Shown are typical examples of EZH2 protein expression in bronchial epithelium, carcinoids and SCLC tumours.

E2F/Rb focused analysis of copy number in SCLC cell lines (n = 14) and tumours (n = 14) revealed gain of at least one of the activating *E2F*s or loss of *RB1* in 14/14 tumour samples and 13/14 cell lines (96% of all samples; 95% CI 80% to 100% ) (Figure 1A). This is concordant with the genomic profiles recently published by Peifer et al [5], which we found to exhibit the same pattern of alteration in 56/63 tumour samples (applying copy number thresholds of <1.7 for genomic loss and >2.3 for genomic gain to the segmented data identified events in 84% of samples, 95% CI 72% to 92%). Although the confidence bound does not include 100% in the Peifer et al study, it is worth noting that in the 29 cases with sequence data, 100% of cases had either *RB1* deletion or a truncating mutation event [5]. This in combination with previous estimates of *RB1* mutation rates in SCLC (60% to 90%) [32], [33] suggests that E2F/RB1 disruption is near universal in SCLC cases.

Real-time PCR analysis of transcript levels demonstrated a striking pattern of E2F activation, as at least two activating E2F members were over expressed by 10× their normal levels in every SCLC cell line, while Rb1 mRNA was highly reduced in most SCLC cell lines (Figure 1B and Table S2). These levels of deregulation are significantly greater than those observed for a panel of NSCLC cell lines based on a one tailed Mann-Whitney U test, supporting the SCLC specific nature of these events (SCLC vs NSCLC: *E2F1* p = 0.0034, *E2F2* p = 2.310×10^–5^, *E2F3* p = 1.744×10^–5^, *Rb1* p = 0.0078; Figure S2). Deregulation of the E2F and Rb1 transcripts is complimentary to the recent results of Byers et al who detected SCLC specific protein level deregulation of Rb and E2F1 in a proteomic profiling study of SCLC, NSCLC and normal lung cell lines [11].

Collectively, it appears that the Rb pathway is deregulated not only through loss of Rb copies/function but also through genomic gains of the E2F transcription factors in SCLC. Taken together, this suggests that the E2F/Rb pathway is activated in all of the SCLC samples in this study, by either loss of *RB1* or copy gain of an *E2F* member gene. Based on our analysis of external data, this holds true in additional clinical SCLC tumour cohorts [5].

### Overexpression of the E2F/Rb Target EZH2

The striking pattern of E2F/Rb deregulation in SCLC cell lines and tumours prompted us to examine genes downstream of the E2F transcription factors. One target of the E2F/Rb pathway which has recently been described as an oncogene in multiple cancer types is *EZH2*. EZH2 is a polycomb group (PcG) gene with a role in embryonic development and differentiation through epigenetic transcriptional regulation of genes critical to maintaining stem-like properties of embryonic cells and establishing lineage specificity [34]–[36]. It directly controls DNA methylation of several target genes including *WNT1*, corroborating previous studies in which multiple genetic hits were observed to shut down the WNT pathway in SCLC cell lines [16], [36]. EZH2 overexpression has been observed in many cancer subtypes including: prostate, breast, bladder, squamous cell lung cancer and hepatocellular carcinomas [37]–[44]. In the case of squamous cell lung cancer, EZH2 expression has been observed in dysplastic squamous cells and tumours but not in normal bronchial epithelial cells, suggesting *EZH2* activation could also be an early event in NSCLC [39]. Additionally, studies of several cancer types have linked expression of EZH2 to aggressiveness, invasion and cisplatin resistance implicating a role for EZH2 in aggressive tumour behaviour [43], [45]–[47].

In our study, expression analysis of EZH2 in NSCLC and SCLC cell lines revealed a striking state of hyperactivation in SCLC compared to NSCLC cells (Mann-Whitney U test, p = 4.52×10^–6^; Figure 1C). Our results suggest that EZH2 is on average, 42-fold overexpressed in SCLC lines compared to 13-fold overexpressed in NSCLC cell lines. To confirm if the overexpression of EZH2 is also present in tumours, we analyzed the data from an independent cDNA expression array study [13], which profiled a separate set of 11 cell lines and a panel of 15 primary SCLC tumours, in addition to 12 adenocarcinomas (NSCLC subtype). Although the exact fold change levels were not directly equivalent (potentially due to differences between RT-PCR and cDNA microarray dynamic range), the trend in expression levels is strikingly similar to our study, with significantly higher expression of EZH2 in SCLC cell lines and tumours compared to NSCLC samples (Figure S2). This is consistent with the results of Byers et al who recently observed increased EZH2 protein expression in SCLC cell lines [11].

To confirm that mRNA levels translate to increased protein levels in clinical tumours, we performed IHC on a tissue array of SCLC tumours, carcinoid and normal lung tissues, and observed increased staining of EZH2 in the SCLC tumour samples (n = 13), compared to carcinoids, and bronchial epithelia (Figure 1D, Table S3). Bracken et al [38] suggested that both direct *E2F* DNA amplification and *RB1* disruption can lead to deregulation of EZH2 expression. The significant EZH2 over-expression we have described in SCLC is likely not due to the low level copy number gains we observe in the SCLC cell lines and tumours, as previous studies have suggested that on average 2-fold copy number gain is associated with an average 1.5-fold change in associated mRNA levels [38], [48], suggesting that over-expression of EZH2 in SCLC, is primarily controlled by E2F/Rb disruption, rather than direct copy number alterations.

To further assess our hypothesis that E2F/Rb disruption leads to EZH2 activation, we evaluated the associations between EZH2 and E2F/RB1 mRNA expression levels in two public SCLC datasets. In a microarray dataset of 56 SCLC lines, we found positive correlations between EZH2 and E2F1 (r^2^ = 0.3957, p = 0.0013) and E2F2 (r^2^ = 0.3124, p = 0.0095), and no significant correlations between EZH2 and E2F3 (r^2^ = 0.1689, p = 0.1067) or RB1 (r^2^ = 0.0712, p = 0.6989) (Table S4). In 15 SCLC tumours with RNA-sequencing data, we observed strong positive correlations between EZH2 and E2F1 (r^2^ = 0.5179, p = 0.0253) and E2F2 (r^2^ = 0.6357, p = 0.0064), and no significant correlations between EZH2 and E2F3 (r^2^ = –0.1571, p = 0.7166) and RB1 (r^2^ = –0.1929, p = 0.2450) (Table S4). The fact that RB1 was not significantly correlated with E2F expression in these datasets likely reflects the low overall expression of RB1 across these samples, and the role of phosphorylation in RB1 function, which is not accounted for in these data. These results provide further evidence that activation of E2F1 and E2F2 is associated with activation of EZH2.

### Effect of E2F Manipulation on EZH2 Expression

We next performed E2F knockdown experiments to directly assess the consequences of E2F modulation on EZH2 expression levels in SCLC. ShRNA-mediated knockdowns of E2F1, E2F2, and E2F3 showed reductions in EZH2 protein levels, fitting with our hypothesis that E2Fs modulate EZH2 expression in SCLC (Figure S3A). Additionally, we performed overexpression experiments in immortalized human bronchial epithelial cells (HBEC) [49], which are a non-malignant model used to study the molecular pathogenesis of non-small cell lung cancer, since to date, there is no non-malignant neuroendocrine model currently available to assess the transforming potential of genes in a SCLC specific precursor cell type. We observed increased expression of EZH2 upon induction of E2F2 (Figure S3B). The effects of E2F knockdown and overexpression on EZH2 levels taken together with the positive correlations we observed between E2F and EZH2 mRNA expression levels suggest that activation of E2Fs leads to a corresponding increase in EZH2 levels. These observations support our hypothesis that increased expression of E2F members leads to EZH2 activation in SCLC.

### PRC2 Targets are Hypermethylated in SCLC

The PcG has been of great interest in many tumour types in recent years, due to its role in transcriptionally silencing expression of tumour suppressor genes via chromatin remodelling and directing DNA methylation. PcG proteins form multi-protein complexes that repress transcription via post-translational modification of histones, specifically tri-methylation of the 27th lysine on histone 3 (H3K27Me3). A high proportion (50–80%) of genes that are aberrantly epigenetically silenced by DNA promoter hypermethylation in most human cancers are those marked by the Polycomb Repressive Complex 2 (PRC2) during embryogenesis, where repressive histone marks function to “turn off” cell fate determining genes, bestowing undifferentiated self-renewal capacities to embryonic stem cells [50]–[53]. Given that EZH2 is the critical catalytic member of the PRC2 complex, and the ability of this complex to recruit the *de novo* DNA methylating enzymes (DNMT3a/b), we were interested in assessing whether CpG promoter methylation in SCLC tumours occurred preferentially in genes marked by PRC2 in embryonic stem cells. Therefore, we analyzed the methylation status of EZH2-PRC2 target genes in our primary tumour samples [22]. Data is available in the Gene Expression Omnibus.

**Figure 2:**
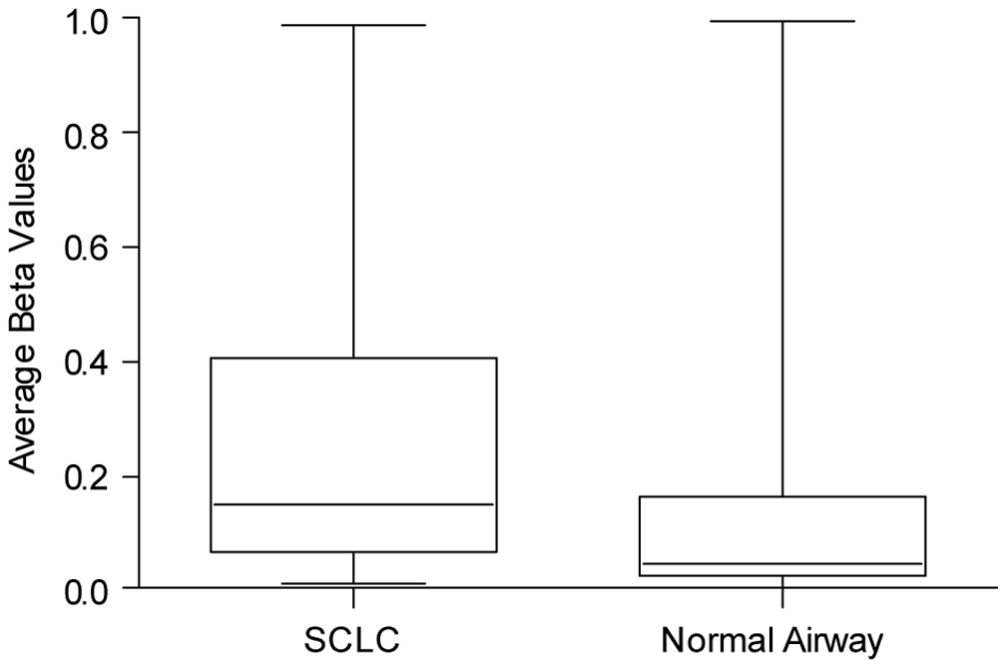
Genes targeted by EZH2 during embryogenesis are hypermethylated in SCLC. Methylation β values for 1683 genes targeted for PRC2 H3KMe3 in mouse embryonic stem cells are plotted for each group. These 1683 genes mapped to 3497 Illumina HM27 probes, of which 18% (640 probes) are hypermethylated and 3% (93 probes) are hypomethylated in SCLC tumours relative to normal airways. Genes targeted for H3KMe3 in mouse embryonic stem cells by EZH2 and the PRC2 complex are preferentially hypermethylated SCLC tumours providing evidence that EZH2 is functionally active in SCLCs.

We found an overwhelming proportion of genes targeted by EZH2 and the PRC2 complex to be hypermethylated in SCLC tumours compared to normal airway epithelia from healthy individuals (Figure 2). After applying stringent filtering criteria, we identified 2756 hypermethylated probes (p value <0.05, delta β> = 0.2), and 1372 hypomethylated probes (p value <0.05, delta β< = –0.2) in SCLC relative to healthy airway epithelial cells. Out of the 3497 probes associated with genes known to be embryonic stem cell EZH2 targets [22], we found 18% (640 probes) of these genes to be hypermethylated, compared to only 3% (93 probes) hypomethylated in SCLC. Strikingly, EZH2-PRC2 target genes accounted for 23% of all hypermethylated probes in these tumours overall. Compared to non-PRC2 targets, enrichment of PRC2 target genes in our hypermethylated probe set was statistically significant (Fisher's exact test, p = 2.2×10^–16^). These data suggest that, analogous to work from several other groups in a variety of cancers [50]–[55], SCLCs display a promoter hypermethylation profile highly concordant with the pattern of genes silenced in embryonic stem cells by the PRC2 complex. We propose that in SCLC, EZH2 overexpression caused by DNA level E2F/Rb pathway disruption results in recruitment of *de novo* DNA methylating enzymes and the establishment of a “stem-cell like” hypermethylator profile.

### EZH2 is Required for Rapid Growth in SCLC Cell Lines

**Figure 3:**
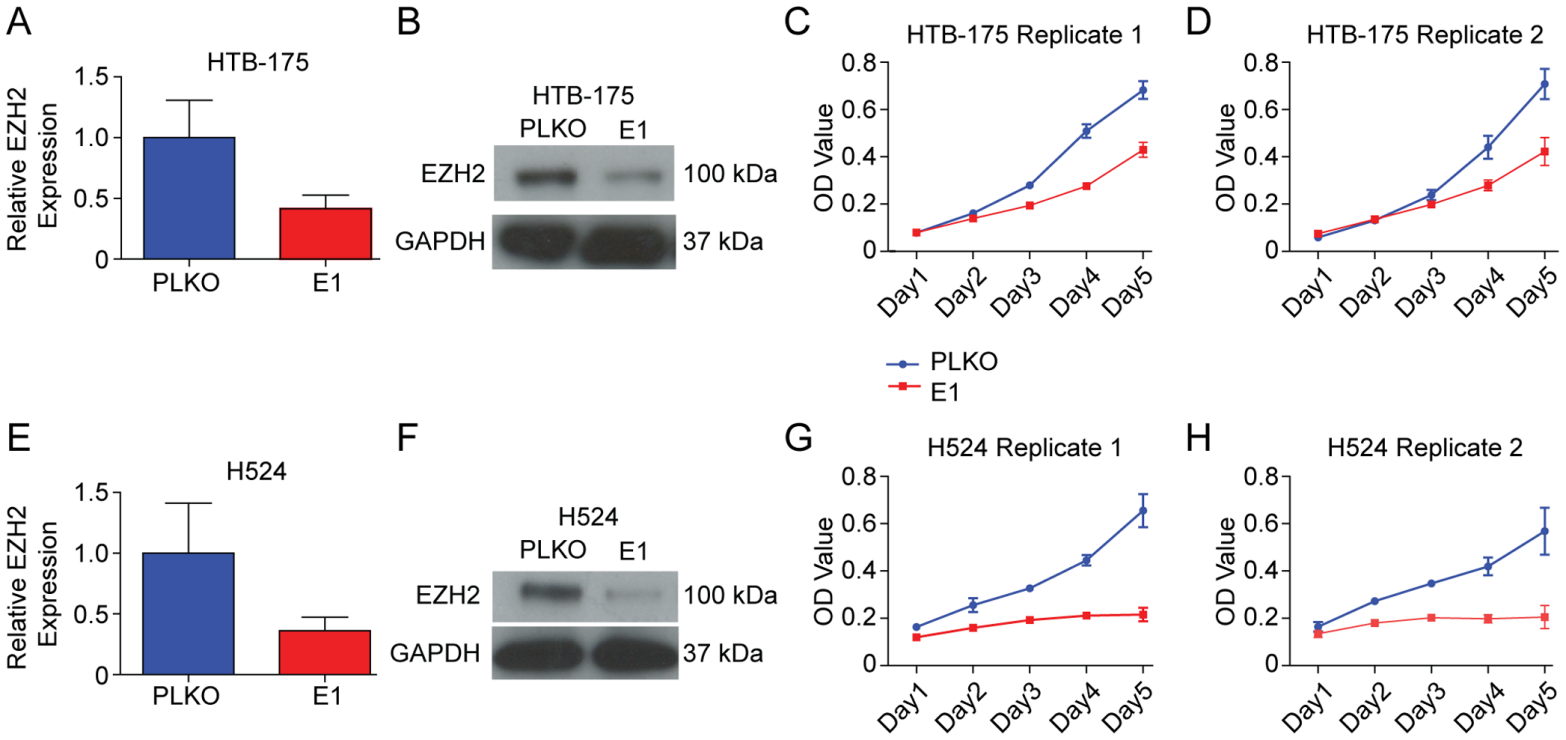
EZH2 knockdown causes a significant reduction in SCLC cell viability. (**A, E**) To confirm knockdown of EZH2, RNA was extracted from the transfected lines and qPCR was performed. Expression in knockdown lines (E1) is shown relative to PLKO controls. (**B,F**) Confirmation of protein levels of EZH2 in the knockdown lines was performed using standard Western blotting methods. Both qPCR and Western blotting verified EZH2 levels were reduced by over 50% in HTB-175 and H524 cells upon shRNA-mediated knockdown. (**C,D,G,H**) To assess the viability of *EZH2* knockdown relative to controls cells we performed MTT assays. Knockdown of EZH2 caused a significant and reproducible decrease in cell viability in both SCLC cell lines (Student's t-test, p<0.05).

We next evaluated the oncogenic potential of EZH2 in SCLC, using lentiviral mediated knockdowns in two SCLC cell lines (H524, HTB175). Both knockdown lines demonstrated a striking reduction in growth compared to empty vector controls (Student's t-test, p<0.05) (Figure 3). Corroborating our findings, a recent study involving proteomic profiling of SCLC cell lines demonstrated overexpression of EZH2 in SCLC and demonstrated reduced growth in H69 cells upon transient siRNA knockdown [11]. The pro-proliferative and anti-differentiation functions of EZH2 are consistent with the highly aggressive and undifferentiated clinical and pathological presentation of SCLC. The strong pro-proliferative nature of EZH2, could also partially explain the tolerance of SCLC cells to such high levels of E2F mRNA, as some E2F proteins are capable of anti-proliferative and pro-apoptotic functions when overexpressed [15], [28]–[30], [38].

### Conclusions

From this work, we propose that the E2F/Rb pathway is disrupted not only through loss or mutation of Rb but also through DNA copy number gains of the E2F transcription factors. We propose that this mechanism drives the specific overexpression of several target genes including EZH2, which results in activation of the PRC2 complex and promotion of aberrant methylation in SCLC. Our findings corroborate previous studies and have strong implications for the treatment of SCLC.

Multiple mitogenic pathways, such as MAPK deregulation are also capable of driving E2F function in the absence of specific upstream hits in SCLC, such as the frequent disruption of EGFR in NSCLC [16], [56]. This may partially explain the activation of multiple autocrine and paracrine mitogenic pathways in SCLC including Hedgehog, Notch and cMET/HGF, which all appear to play important roles in disease development [6], [27], [57]–[60]. Thus, it is likely that the best targets for therapeutic intervention in SCLC will be the downstream pathway components, such as EZH2 in the E2F/Rb pathway, which directly affect SCLC phenotypes including rapid cell division and resistance to therapy.

Our results demonstrating the hyperactivation of EZH2 through recurrent DNA level E2F/Rb deregulation; the high protein expression in SCLC tumours; and absence of protein expression in normal lung parenchyma, taken together with the oncogenic properties of EZH2 and associated aberrant methylation profiles in SCLCs, confirm the notion that EZH2 should be prioritized as a target for therapeutic intervention for SCLC treatment.

## Supporting Information

Figure S1 **Array CGH profiling of SCLC tumours.** Comparison of SCLC tumour and cell lines genomes. Alteration frequencies for SCLC tumours (red) and cell lines (green) are displayed as bar plots adjacent to chromosomal ideograms. Bars extending to the right and left of the chromosome represent regions of gain and loss respectively, with yellow representing regions of overlap. Vertical bars on the left of the frequency diagrams represent SCLC specific regions identified in a previous cell line study, with green and red shading representing SCLC specific loss and gain. Tissue cores were used in lieu of microdissection due to the high purity of the SCLC samples. Clinical details of the samples are summarized in Table S1. In order to identify genomic regions involved in the tumourigenesis of SCLC we analyzed these 14 tumour samples using the SMRT CGH array which is robust to FFPE DNA. Initial analysis of the DNA profiles for the SCLC tumours revealed the presence of many known regions of copy number alteration in lung cancer, such as loss of 3p and gain of 5p. To determine which regions may be relevant to the SCLC phenotype, we examined the tumour CGH profiles in the context of a panel of 14 SCLC cell lines which we previously analysed in comparison to NSCLC cell lines to identify SCLC specific regions of copy number alteration. We hypothesized that SCLC specific regions from our cell line analysis that are validated in primary tumours represent genomic loci key to the aggressive phenotype observed in clinical disease. In general, SCLC tumours tend to display fewer regions of frequent alteration compared to cell lines and several regions demonstrate different patterns of alteration. It is likely that a primary reason for observing more genomic alterations in cell lines is related to the differences in the sample populations. The cell lines mostly reflect advanced disease and have likely acquired alterations due to growth in culture, while the tumours reflect mostly limited disease lesions (9/14); thus, we expect that patterns of alteration will differ at some regions of the genome due to cell culture induced alterations or markers of advanced disease. Comparison of the tumour and cell line profiles revealed highly complementary frequency profiles with similar alteration frequencies at 1p, 3q, 5q, 10q, and 18q.
(DOC)

Figure S2 **EZH2 is hyperactivated in SCLC compared to NSCLC.** Significant overexpression of EZH2 in SCLC compared to NSCLC samples, observed in an independent data set consisting of SCLC tumours and cell lines with adenocarcinma representing NSCLC.
(DOC)

Figure S3 **Effects of E2F manipulation on EZH2 levels in H524 and HBEC cells.** Western blots depicting the effects of shRNA mediated knockdown (A) and overexpression (B) of E2F1, E2F2, and E2F3 on EZH2 levels in H524 and HBEC cells, respectively. Band intensities corresponding to protein expression levels were normalized to each respective loading control (GAPDH or Histone H3, HH3), and the proportion of expression in each modified line (knockdown or overexpression) relative to the control line is indicated.
(DOC)

Table S1 **Clinical features of SCLC tumours.** Summary of clinical features for the SCLC tumour samples analyzed in this study.
(DOC)

Table S2 **Real time PCR analysis of E2F/Rb family members.** Expression levels of E2F1, E2F2, E2F3, and RB1 in SCLC cell lines as assessed by real time quantitative PCR.
(DOC)

Table S3 **Immunohistochemistry results.** Summary of EZH2 staining intensities for SCLC and carcinoid tissues.
(DOC)

Table S4 **Correlation between EZH2 and E2F/Rb expression levels.** Summary of correlations between EZH2 and E2F/Rb family members in external cohorts of SCLC cell lines and tumours.
(DOCX)
