# Supplementary material for: Genomic Deregulation of the E2F/Rb Pathway Leads to Activation of the Oncogene EZH2 in Small Cell Lung Cancer
Source: PLoS One. 2013 Aug 15;8(8):e71670. doi: 10.1371/journal.pone.0071670 (PMC3744458; doi:10.1371/journal.pone.0071670)
Supplement: Figure S1 — Array CGH profiling of SCLC tumours. Comparison of SCLC tumour and cell lines genomes. Alteration frequencies for SCLC tumours (red) and cell lines (green) are displayed as bar plots adjacent to chromosomal ideograms. Bars extending to the right and left of the chromosome represent regions of gain and loss respectively, with yellow representing regions of overlap. Vertical bars on the left of the frequency diagrams represent SCLC specific regions identified in a previous cell line study, with green and red shading representing SCLC specific loss and gain. Tissue cores were used in lieu of microdissection due to the high purity of the SCLC samples. Clinical details of the samples are summarized in Table S1. In order to identify genomic regions involved in the tumourigenesis of SCLC we analyzed these 14 tumour samples using the SMRT CGH array which is robust to FFPE DNA. Initial analysis of the DNA profiles for the SCLC tumours revealed the presence of many known regions of copy number alteration in lung cancer, such as loss of 3p and gain of 5p. To determine which regions may be relevant to the SCLC phenotype, we examined the tumour CGH profiles in the context of a panel of 14 SCLC cell lines which we previously analysed in comparison to NSCLC cell lines to identify SCLC specific regions of copy number alteration. We hypothesized that SCLC specific regions from our cell line analysis that are validated in primary tumours represent genomic loci key to the aggressive phenotype observed in clinical disease. In general, SCLC tumours tend to display fewer regions of frequent alteration compared to cell lines and several regions demonstrate different patterns of alteration. It is likely that a primary reason for observing more genomic alterations in cell lines is related to the differences in the sample populations. The cell lines mostly reflect advanced disease and have likely acquired alterations due to growth in culture, while the tumours reflect mostly limited disease [file pone.0071670.s001.doc]

**Figure S1**

**Figure S1.** **Array CGH Profiling of SCLC Tumours.**
