# Supplementary material for: Genomic Deregulation of the E2F/Rb Pathway Leads to Activation of the Oncogene EZH2 in Small Cell Lung Cancer
Source: PLoS One. 2013 Aug 15;8(8):e71670. doi: 10.1371/journal.pone.0071670 (PMC3744458; doi:10.1371/journal.pone.0071670)
Supplement: Figure S2 — EZH2 is hyperactivated in SCLC compared to NSCLC. Significant overexpression of EZH2 in SCLC compared to NSCLC samples, observed in an independent data set consisting of SCLC tumours and cell lines with adenocarcinma representing NSCLC. (DOC) [file pone.0071670.s002.doc]

**Figure S2**

**Figure S2. EZH2 is hyperactivated in SCLC compared to NSCLC**.
