# Supplementary figures and images for: Genomic Deregulation of the E2F/Rb Pathway Leads to Activation of the Oncogene EZH2 in Small Cell Lung Cancer
Source: PLoS One. 2013 Aug 15;8(8):e71670. doi: 10.1371/journal.pone.0071670 (PMC3744458; doi:10.1371/journal.pone.0071670)

**Figure S3**


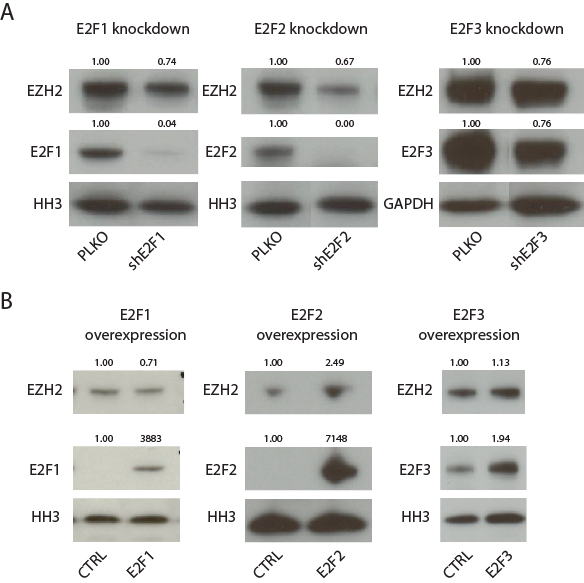


**Figure S3. Effects of E2F manipulation on EZH2 levels in H524 and HBEC cells.**

Supplement: Figure S3 — Effects of E2F manipulation on EZH2 levels in H524 and HBEC cells. Western blots depicting the effects of shRNA mediated knockdown (A) and overexpression (B) of E2F1, E2F2, and E2F3 on EZH2 levels in H524 and HBEC cells, respectively. Band intensities corresponding to protein expression levels were normalized to each respective loading control (GAPDH or Histone H3, HH3), and the proportion of expression in each modified line (knockdown or overexpression) relative to the control line is indicated. (DOC) [file pone.0071670.s003.doc]
