# Supplementary material for: Genomic Deregulation of the E2F/Rb Pathway Leads to Activation of the Oncogene EZH2 in Small Cell Lung Cancer
Source: PLoS One. 2013 Aug 15;8(8):e71670. doi: 10.1371/journal.pone.0071670 (PMC3744458; doi:10.1371/journal.pone.0071670)
Supplement: Table S1 — Clinical features of SCLC tumours. Summary of clinical features for the SCLC tumour samples analyzed in this study. (DOC) [file pone.0071670.s004.doc]

**Table S1: Clinical features of SCLC t**umours

| **ID** | **Diagnosis** | **Sex** | **Age** | **Limited vs Extensive Disease (metastisis)** | **Comment** |
| --- | --- | --- | --- | --- | --- |
| **sc-35** | SCLC | M | 59 | E (other) |  |
| **sc-37** | SCLC | F | 63 | E (bone) |  |
| **sc-65** | SCLC | F | 74 | L |  |
| **sc-68** | SCLC | M | 69 | L |  |
| **sc-73** | SCLC | F | 42 | L | The sample is after 2nd course of Chemotherapy, History of other neoplasms |
| **sc-74** | SCLC | M | 77 | L |  |
| **sc-75** | SCLC | F | 78 | L | RLL wedge resection SQCC + SCLC Sample just from SCLC History of other neoplasms |
| **sc-76** | SCLC | M | 70 | E | History of other neoplasms |
| **sc-77** | SCLC | F | 62 | L |  |
| **sc-78** | SCLC | F | 75 | L |  |
| **sc-79B** | SCLC (combined tumor) | F | 57 | E |  |
| **sc-80** | SCLC | F | 64 | L |  |
| **sc-81** | SCLC | F | 71 | E |  |
| **sc-82A** | SCLC (combined tumor) | M | 80 | L |  |
