# Supplementary material for: Genomic Deregulation of the E2F/Rb Pathway Leads to Activation of the Oncogene EZH2 in Small Cell Lung Cancer
Source: PLoS One. 2013 Aug 15;8(8):e71670. doi: 10.1371/journal.pone.0071670 (PMC3744458; doi:10.1371/journal.pone.0071670)
Supplement: Table S2 — Real time PCR analysis of E2F/Rb family members. Expression levels of E2F1, E2F2, E2F3, and RB1 in SCLC cell lines as assessed by real time quantitative PCR. (DOC) [file pone.0071670.s005.doc]

**Table S2: Real time PCR analysis of E2F/Rb family m**embers

|  |  | **Absolute Expression (0-1000)** | | | | **Relative Expression (Sample/Normal Lung)** | | | |
| --- | --- | --- | --- | --- | --- | --- | --- | --- | --- |
| **Sample** | **RB1 Protein** | **E2F1** | **E2F2** | **E2F3** | **RB1** | **E2F1** | **E2F2** | **E2F3** | **RB1** |
| Normal |  | 1.223 | 0.001 | 1.405 | 0.703 | 1.000 | 1.000 | 1.000 | 1.000 |
| H187 | -ve | 508.740 | 0.285 | 1.405 | 0.002 | 415.873 | 445.722 | 1.000 | 0.003 |
| H841 | +ve | 57.313 | 0.064 | 0.306 | 0.703 | 46.851 | 100.427 | 0.218 | 1.000 |
| H378 | nd | 179.867 | 0.266 | 12.913 | 0.001 | 147.033 | 415.873 | 9.190 | 0.002 |
| H1607 | -ve | 173.740 | 0.306 | 86.870 | 5.819 | 142.025 | 477.713 | 61.820 | 8.282 |
| H889 | -ve | 34.674 | 0.156 | 3.906 | 0.001 | 28.345 | 243.032 | 2.780 | 0.001 |
| H289 | -ve | 83.911 | 0.865 | 3.118 | 0.079 | 68.594 | 1351.176 | 2.219 | 0.113 |
| HCC33 | -ve | 37.163 | 0.236 | 4.487 | 0.106 | 30.379 | 368.367 | 3.193 | 0.151 |
| H2171 | -ve | 20.978 | 0.023 | 2.715 | 0.085 | 17.148 | 35.506 | 1.932 | 0.121 |
| H82 | -ve | 138.696 | 0.220 | 15.625 | 0.093 | 113.378 | 343.699 | 11.119 | 0.132 |
| H2141 | -ve | 413.225 | 0.960 | 13.369 | 0.209 | 337.794 | 1499.224 | 9.514 | 0.297 |
| H1672 | -ve | 564.482 | 0.069 | 8.229 | 0.143 | 461.440 | 107.635 | 5.856 | 0.203 |
| H526 | -ve | 385.553 | 0.753 | 10.489 | 0.679 | 315.173 | 1176.267 | 7.464 | 0.966 |
| H524 | -ve | 1000.000 | 3.401 | 16.746 | 0.059 | 817.458 | 5311.855 | 11.917 | 0.084 |
| H2107 | nd | 366.021 | 1.084 | 7.041 | 0.150 | 299.207 | 1692.570 | 5.011 | 0.214 |
