# Supplementary material for: Genomic Deregulation of the E2F/Rb Pathway Leads to Activation of the Oncogene EZH2 in Small Cell Lung Cancer
Source: PLoS One. 2013 Aug 15;8(8):e71670. doi: 10.1371/journal.pone.0071670 (PMC3744458; doi:10.1371/journal.pone.0071670)
Supplement: Table S3 — Immunohistochemistry results. Summary of EZH2 staining intensities for SCLC and carcinoid tissues. (DOC) [file pone.0071670.s006.doc]

**Table S3: Immunohistochemistry results**

|  | **Staining Intensity** | | |
| --- | --- | --- | --- |
| **Samples** | **1** | **2** | **3** |
| **Small Cell Carcinoma** | 0 | 1 | 12 |
| **Carcinoid** | 8 | 13 | 3 |
