# Supplementary material for: Genomic Deregulation of the E2F/Rb Pathway Leads to Activation of the Oncogene EZH2 in Small Cell Lung Cancer
Source: PLoS One. 2013 Aug 15;8(8):e71670. doi: 10.1371/journal.pone.0071670 (PMC3744458; doi:10.1371/journal.pone.0071670)
Supplement: Table S4 — Correlation between EZH2 and E2F/Rb expression levels. Summary of correlations between EZH2 and E2F/Rb family members in external cohorts of SCLC cell lines and tumours. (DOCX) [file pone.0071670.s007.docx]

**Table S4. Correlation between EZH2 and E2F/Rb expression levels**

|  | **External Cohort** | |
| --- | --- | --- |
| **Gene** | **Sanger SCLC Lines (n=56)** | **Peifer et al. Tumours (n=15)** |
| **E2F1** | 0.3957 | 0.5179 |
| **E2F2** | 0.3124 | 0.6357 |
| **E2F3** | 0.1689 | -0.1571 |
| **RB1** | 0.07116 | -0.1929 |
